# Supplementary material for: Metformin Treatment Reduces CRC Aggressiveness in a Glucose-Independent Manner: An In Vitro and Ex Vivo Study
Source: Cancers (Basel). 2023 Jul 22;15(14):3724. doi: 10.3390/cancers15143724 (PMC10378121; doi:10.3390/cancers15143724)
Supplement: Supplementary file 1 [file cancers-15-03724-s001.zip › cancers-2343434-supplementary.pdf]

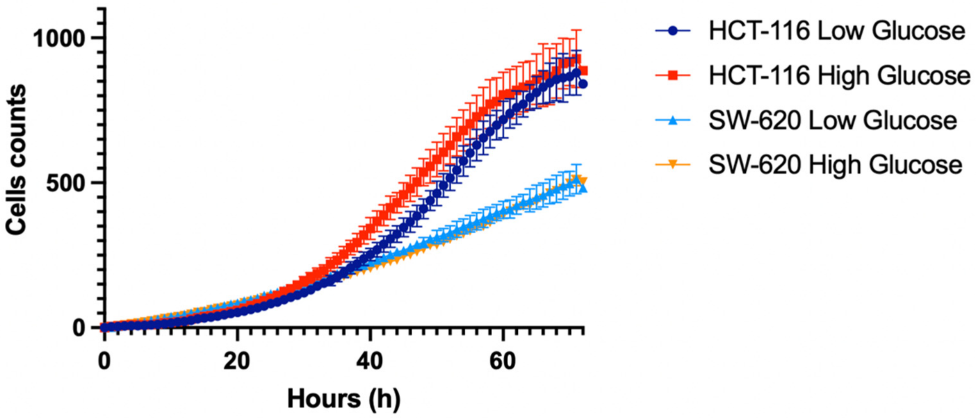

Figure S1 : Growth curve of HCT-116 and SW-620 in Low and High Glucose. Cell count measurement over 72 hours with the Incucyte and the cell by cell software (Sartorius). Made in technical duplicate.

(a)

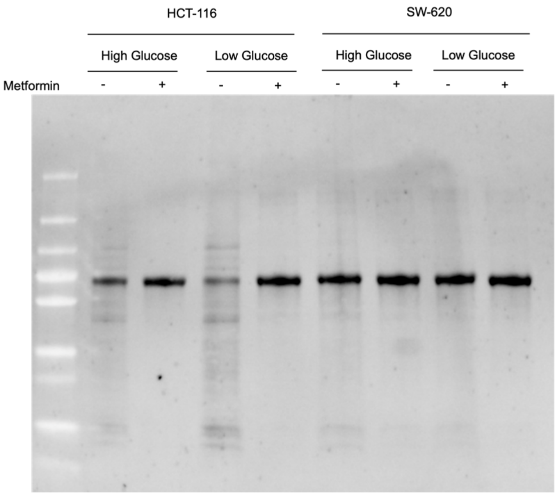

(b)

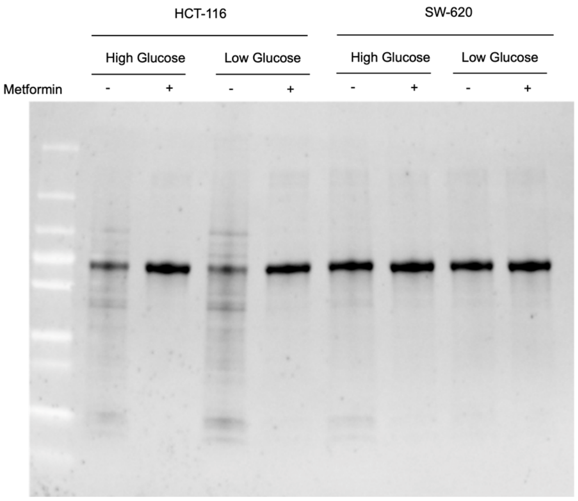

(c)

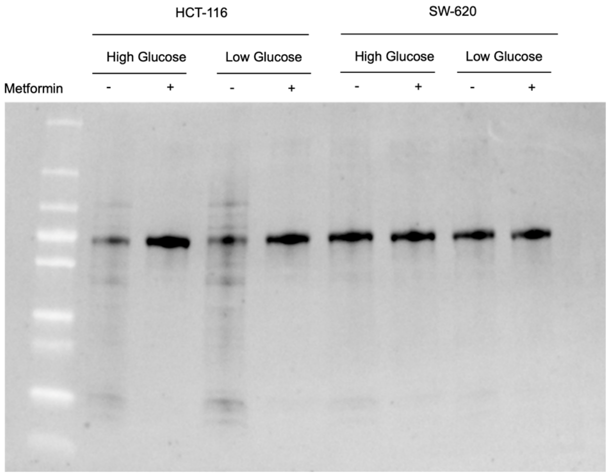

(d)

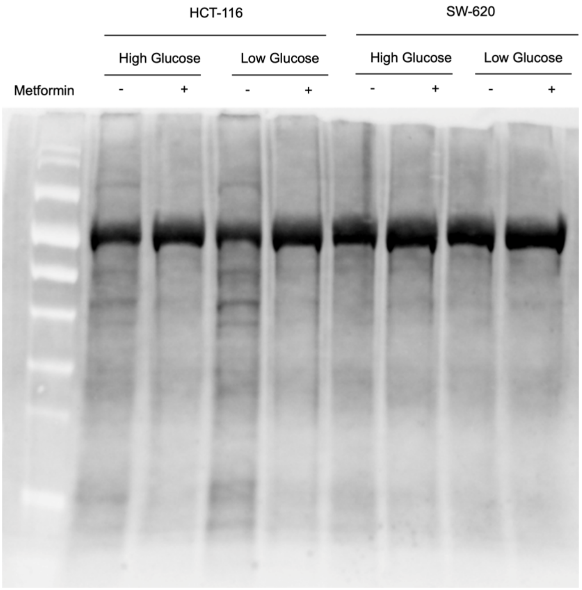

Figure S2 : Quantification of total protein with QStain Total Western Blot ***in vitro*** **Figure 3. (a)** Western Blot E-cadherin **(b)** Western Blot Sortilin **(c)** Western Blot LC3-B **(d)** Western Blot MMP2 / MMP9

(a)

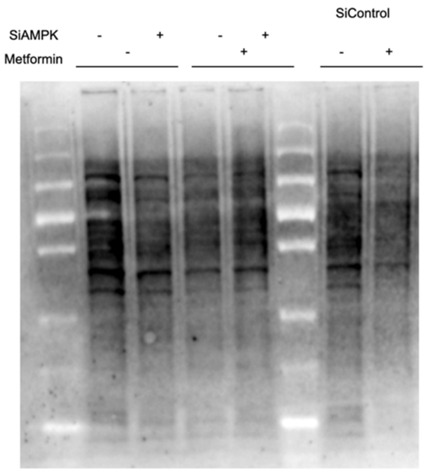
SiControl
SiAMPK - + - +
Metformin - + - +

(b)

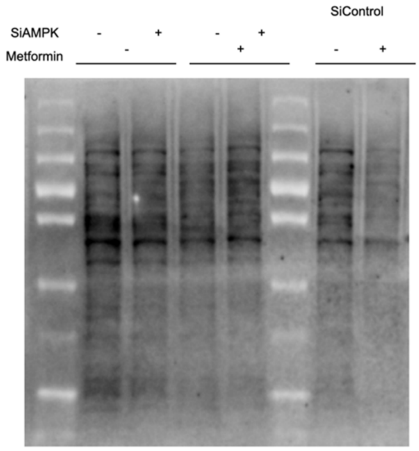
SiControl
SiAMPK - + - +
Metformin - + - +

(c)

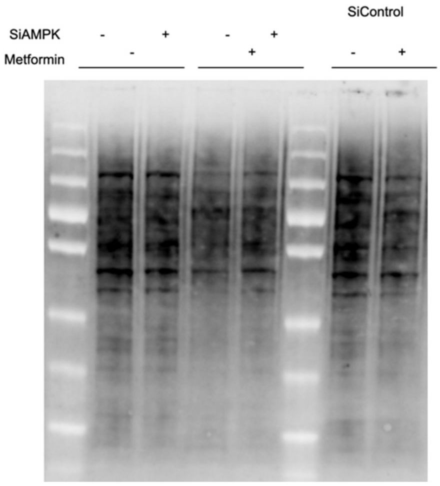
SiControl
SiAMPK - + - +
Metformin - + - +

(d)

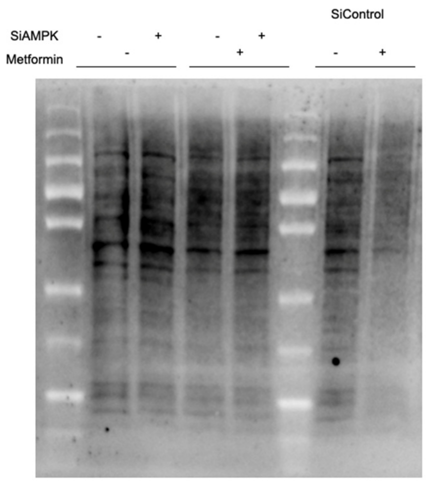
SiControl
SiAMPK - + - +
Metformin - + - +

(e)

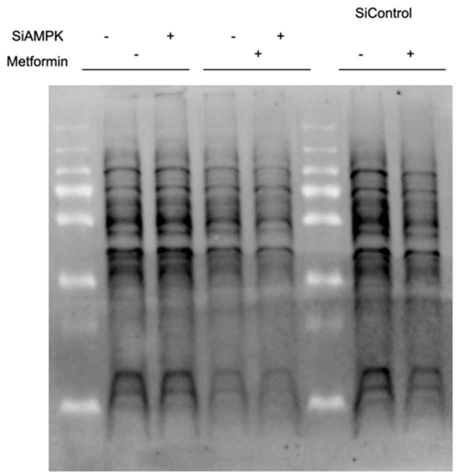
SiControl
SiAMPK - + - +
Metformin - + - +

(f)

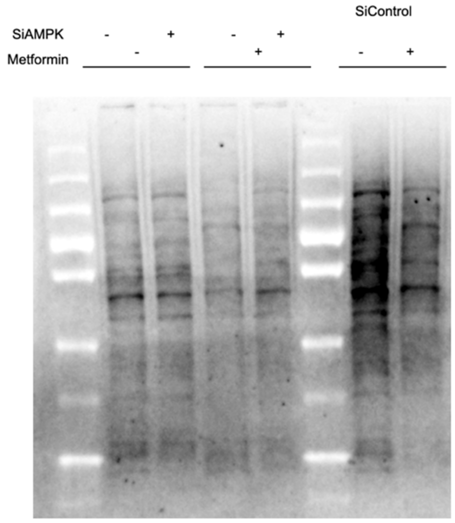
SiControl
SiAMPK - + - +
Metformin - + - +

(g)

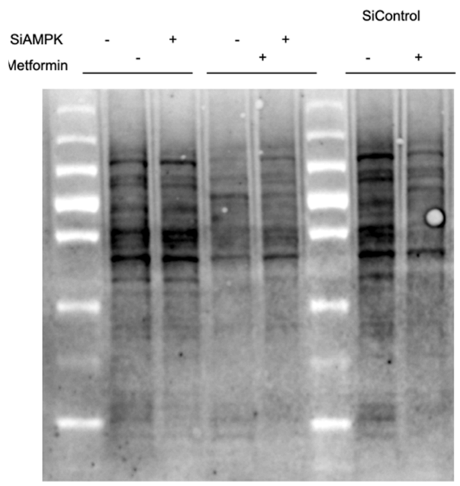
SiControl
SiAMPK - + - +
letformin - + - +

(h)

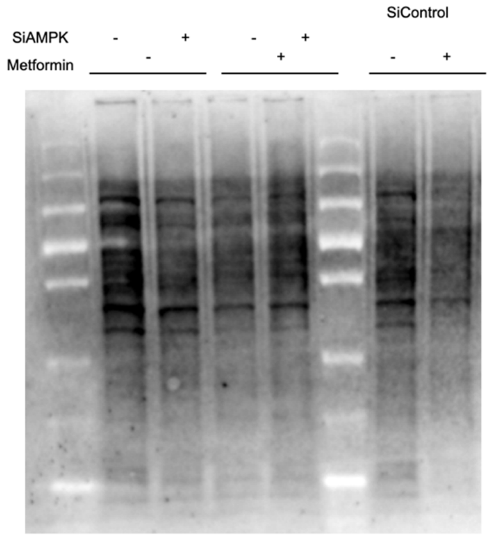
SiControl
SiAMPK - + - +
Metformin - + - +

(i)

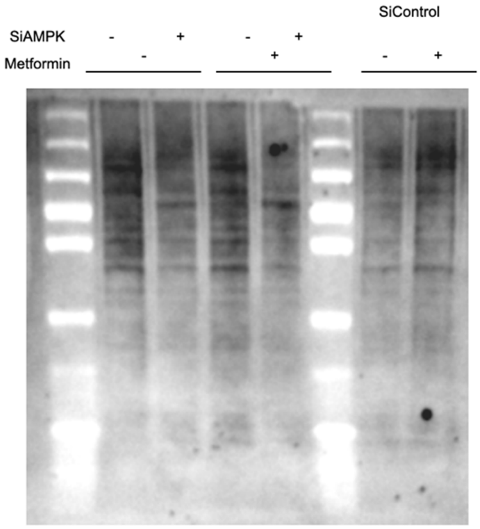
SiControl
SiAMPK - + - +
Metformin - + - +

(j)

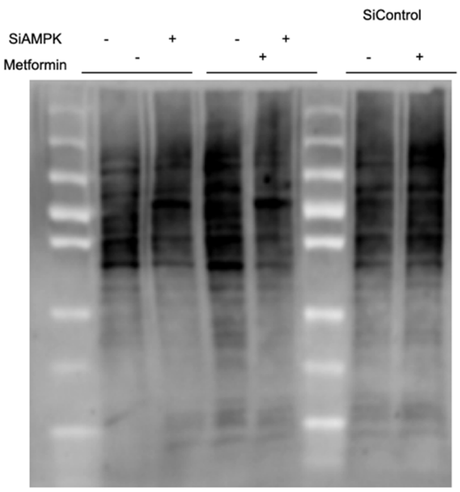
SiControl
SiAMPK - + - +
Metformin - + - +

(k)

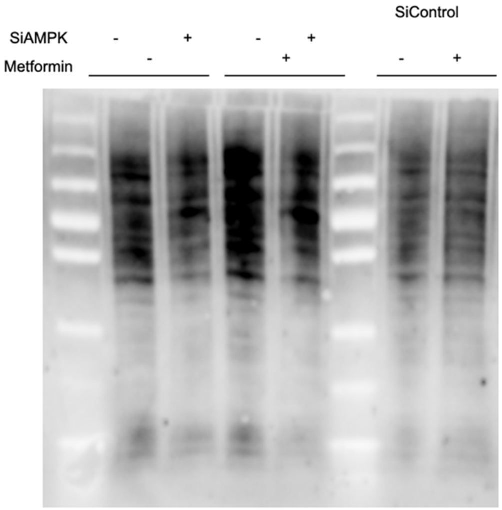
SiControl
SiAMPK - + - +
Metformin - + - +

(l)

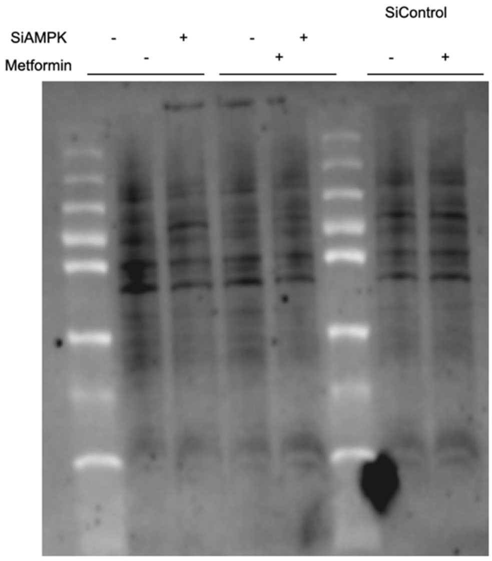
SiControl
SiAMPK - + - +
Metformin - + - +

(m)

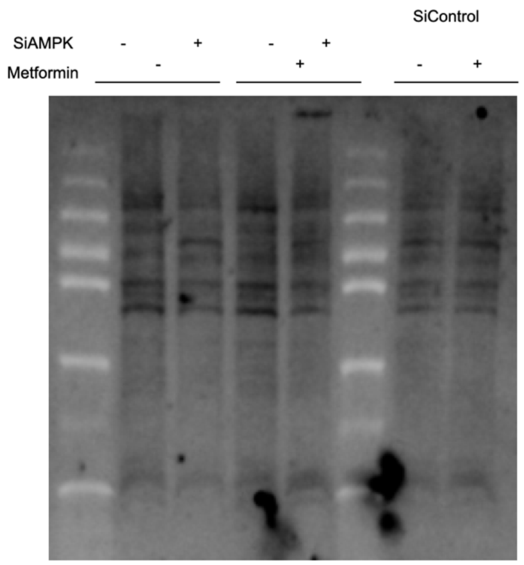

(n)

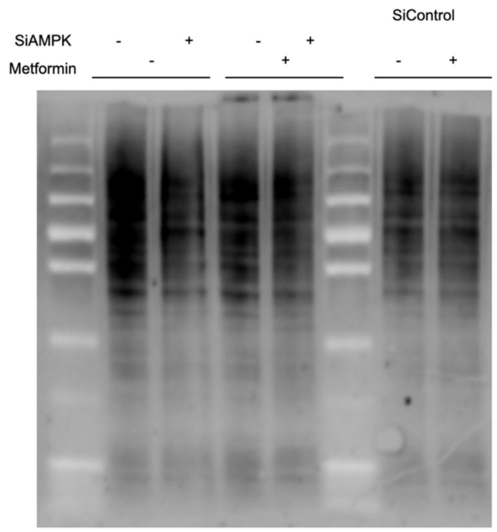

Figure S3 : Quantificaton of total protein with QStain Total Western Blot *in vitro* Figure 4. (a) Western Blot AMPK/Sortilin HCT-116 High-Glucose **(b)** Western Blot E-cadherin HCT-116 High- Glucose **(c)** Western Blot MMP2/MMP9 HCT-116 High- Glucose **(d)** Western Blot LC3-B HCT-116 High- Glucose **(e)** Western Blot AMPK HCT-116 Low-Glucose **(f)** Western Blot E-cadherin HCT-116 Low-Glucose **(g)** Western Blot MMP2/MMP9/LC3-B HCT-116 Low-Glucose **(h)** Western Blot Sortilin HCT-116 Low-Glucose **(i)** Western Blot AMPK/Sortilin SW-620 High-Glucose **(j)** Western Blot E-cadherin SW-620 High-Glucose **(k)** Western Blot MMP2/MMP9/LC3-B SW-620 High-Glucose **(l)** Western Blot AMPK/Sortilin SW-620 Low-Glucose **(m)** Western Blot E-cadherin SW-620 Low-Glucose **(n)** Western Blot MMP2/MM9/LC3-B SW-620 Low-Glucose

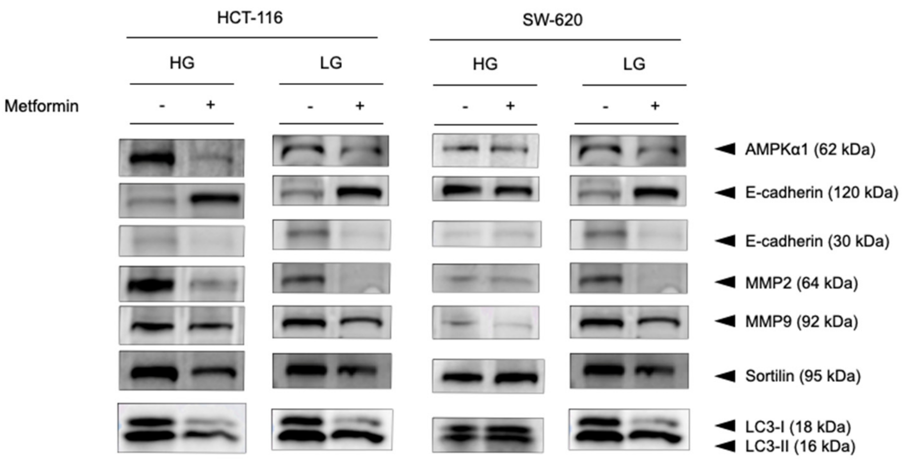

Figure S4 : Western Blot of siRNA Control used in reference to siRNA AMPK presented in Figure 4.

(a)

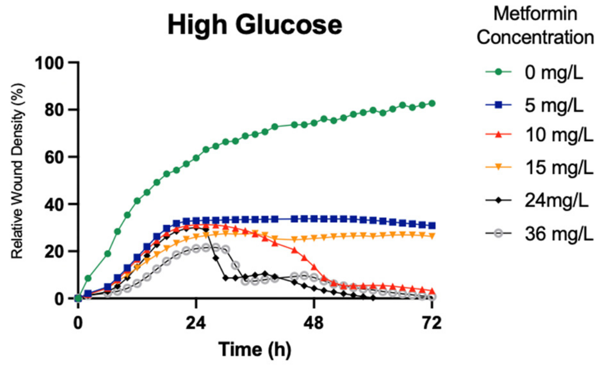

(b)

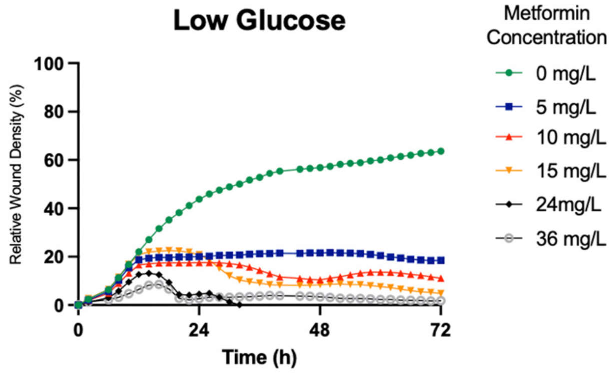

Figure S5 : Impact of Metformin on HCT-116 migration depending on glucose concentrations. (**a**) HCT-116 cells in High Glucose media were treated with different doses of Metformin for 72 hours. Relative Wound Density was

quantified by wound-healing assay using Incucyte software. (**b**) HCT-116 cells in Low Glucose media were treated with different doses of Metformin for 72 hours. Relative Wound Density was quantified by wound-healing assay using Incucyte software.
