# Supplementary material for: Metformin Treatment Reduces CRC Aggressiveness in a Glucose-Independent Manner: An In Vitro and Ex Vivo Study
Source: Cancers (Basel). 2023 Jul 22;15(14):3724. doi: 10.3390/cancers15143724 (PMC10378121; doi:10.3390/cancers15143724)
Supplement: Supplementary file 1 [file cancers-15-03724-s001.zip › File S1. Western Blot Boutaud et al. .pdf]

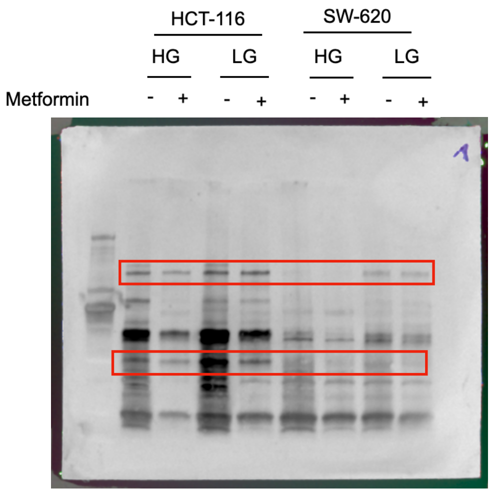

Western Blot : E-cadherin *in vitro*
Figure 3(b)

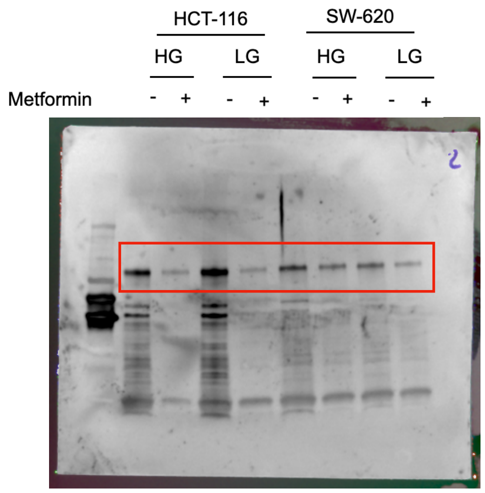

Western Blot : Sortilin *in vitro*
Figure 3(b)

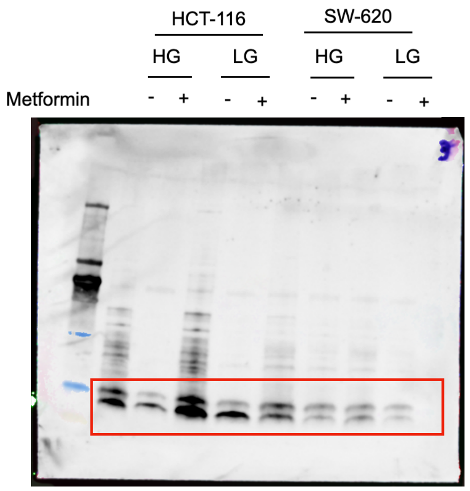

Western Blot : LC3-B *in vitro*
Figure 3(b)

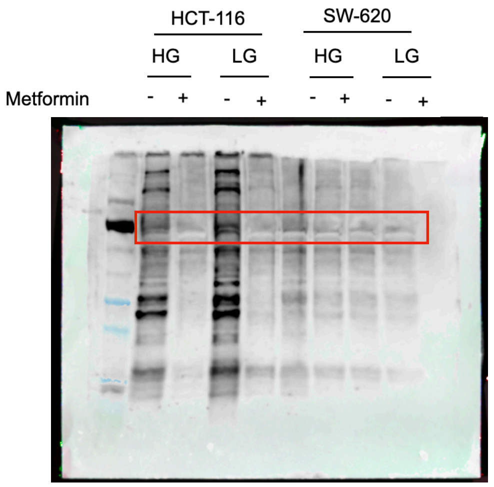

Western Blot : MMP2 *in vitro*
Figure 3(b)

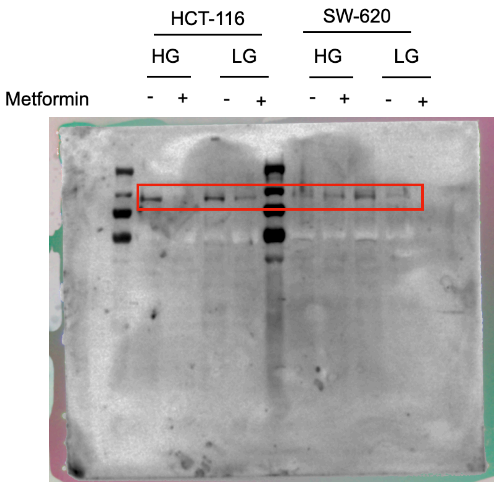

Western Blot : MMP9 *in vitro*
Figure 3(b)

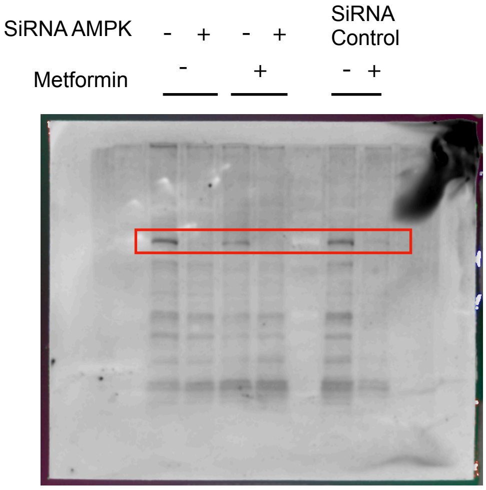

Western Blot : AMPK HCT-116 High Glucose *in vitro*
Figure 4(b)

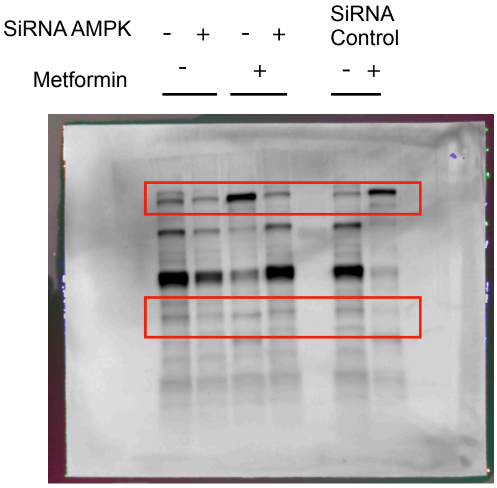

Western Blot : E-cadherin HCT-116 High Glucose *in vitro*
Figure 4(b)

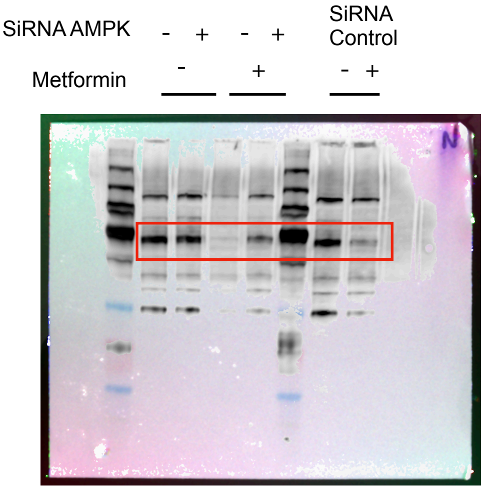

Western Blot : MMP2 HCT-116 High Glucose *in vitro*
Figure 4(b)

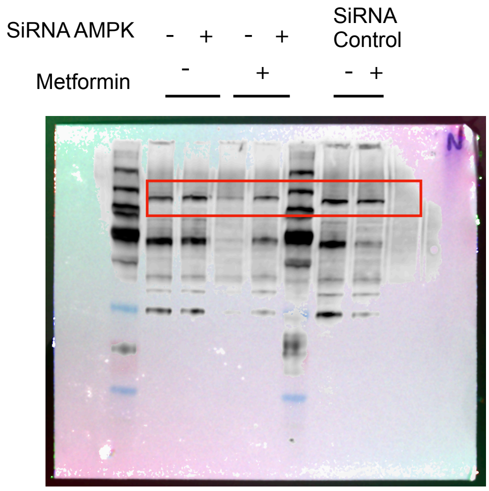

Western Blot : MMP9 HCT-116 High Glucose *in vitro*
Figure 4(b)

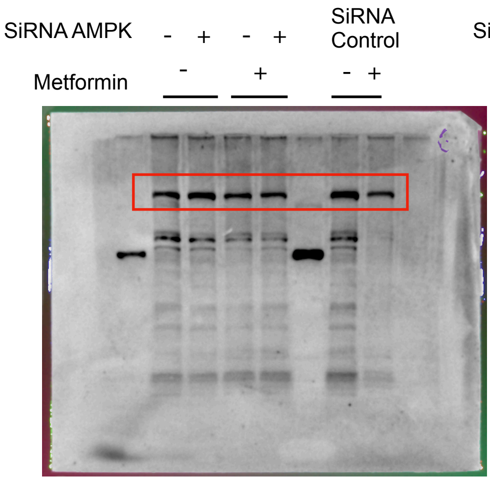

Western Blot : Sortilin HCT-116 High Glucose *in vitro*
Figure 4(b)

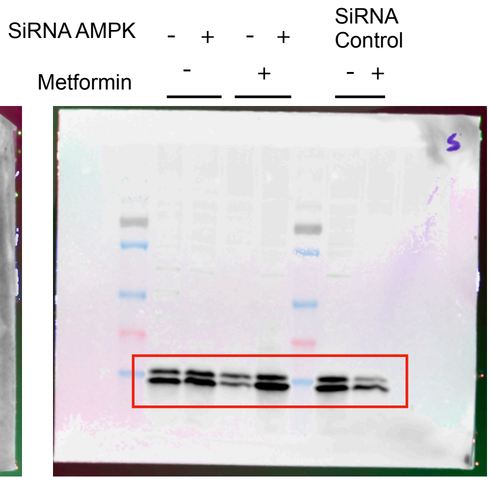

Western Blot : LC3-B HCT-116 High Glucose *in vitro*
Figure 4(b)

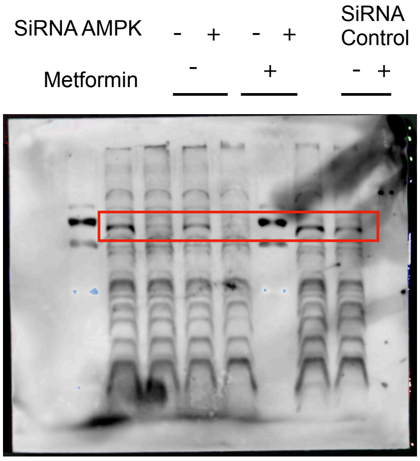

Western Blot : AMPK HCT-116 Low Glucose *in vitro*
Figure 4(a)

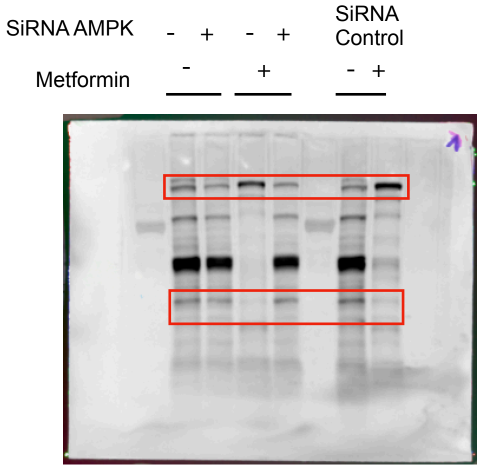

Western Blot : E-cadherin HCT-116 Low Glucose *in vitro*
Figure 4(a)

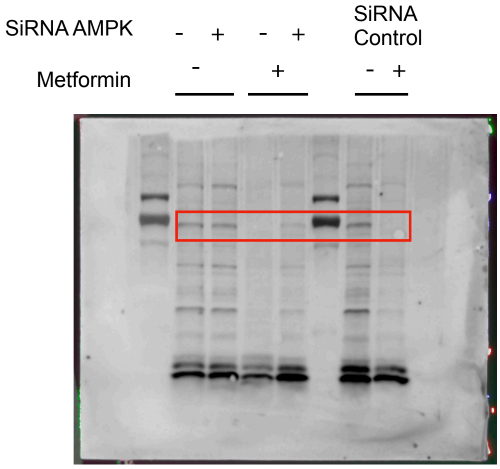

Western Blot : MMP2 HCT-116 Low Glucose *in vitro*
Figure 4(a)

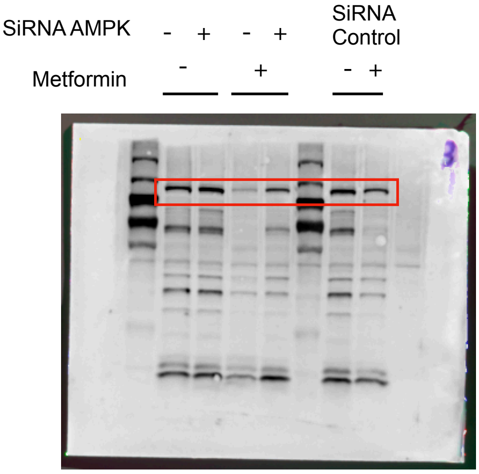

Western Blot : MMP9 HCT-116 Low Glucose *in vitro*
Figure 4(a)

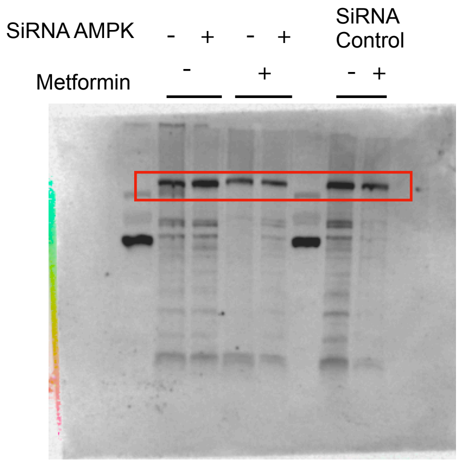

Western Blot : Sortilin HCT-116 Low Glucose *in vitro*
Figure 4(a)

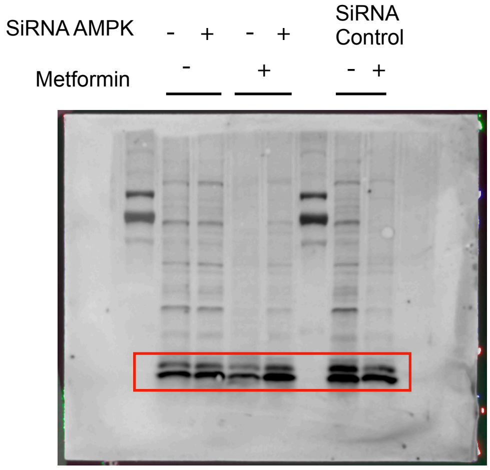

Western Blot : LC3-B HCT-116 Low Glucose *in vitro*
Figure 4(a)

SiRNA AMPK - + - + SiRNA Control

Metformin - + - +

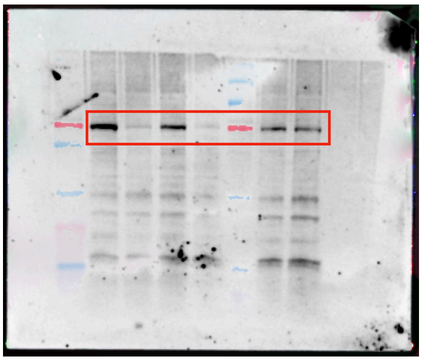

Western Blot : AMPK SW-620 High Glucose *in vitro*
Figure 4(f)

SiRNA AMPK - + - + SiRNA Control

\- + - +

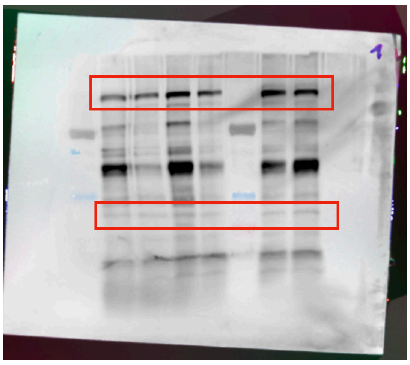

Western Blot : E-cadherin SW-620 High Glucose *in vitro*
Figure 4(f)

SiRNA AMPK - + - + SiRNA Control

\- + - +

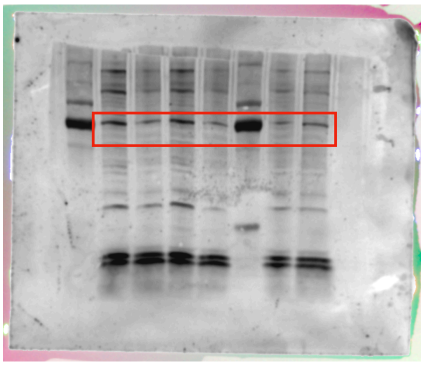

Western Blot : MMP2 SW-620 High Glucose *in vitro*
Figure 4(f)

SiRNA AMPK - + - + SiRNA Control

Metformin - + - +

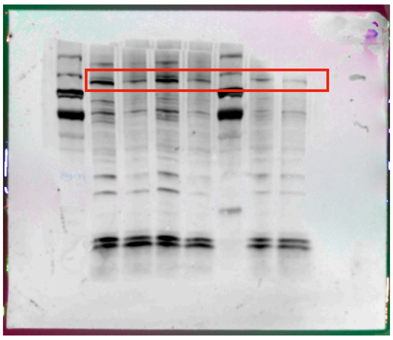

Western Blot : MMP9 SW-620 High Glucose *in vitro*
Figure 4(f)

SiRNA AMPK - + - + SiRNA Control

\-

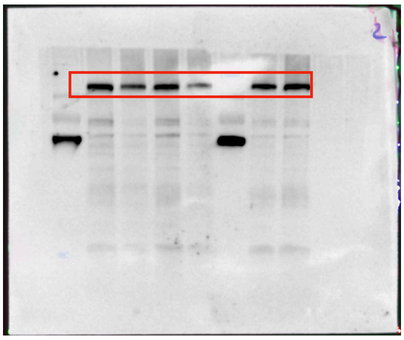

Western Blot : Sortilin SW-620 High Glucose *in vitro*
Figure 4(f)

SiRNA AMPK - + - + SiRNA Control

Metformin - + - +

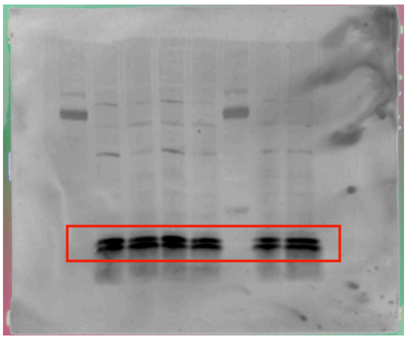

Western Blot : LC3-B SW-620 High Glucose *in vitro*
Figure 4(f)

SiRNA AMPK - + - + SiRNA Control

Metformin - + - +

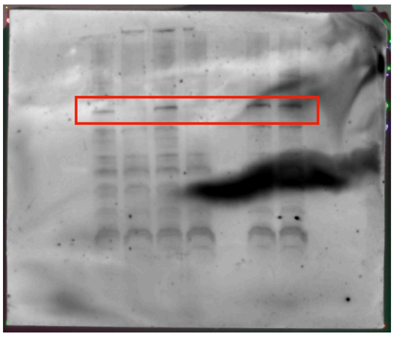

Western Blot : AMPK SW-620 Low Glucose *in vitro*
Figure 4(e)

SiRNA AMPK - + - + SiRNA Control

Metformin - + - +

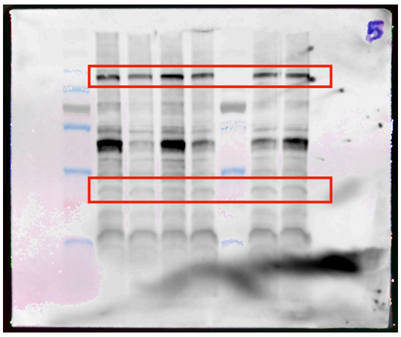

Western Blot : E-cadherin SW-620 Low Glucose *in vitro*
Figure 4(e)

SiRNA AMPK - + - + SiRNA Control

Metformin - + - +

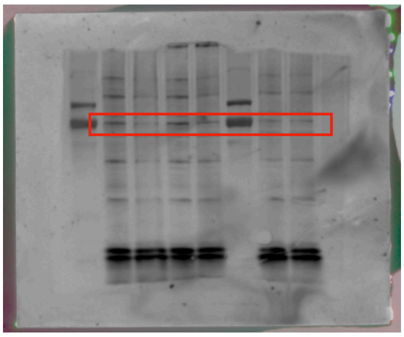

Western Blot : MMP2 SW-620 Low Glucose *in vitro*
Figure 4(e)

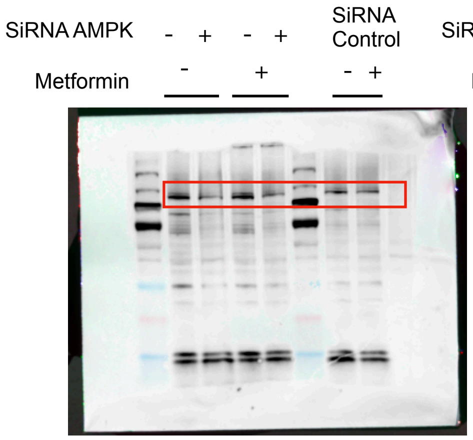

Western Blot : MMP9 SW-620 Low Glucose *in vitro*
Figure 4(e)

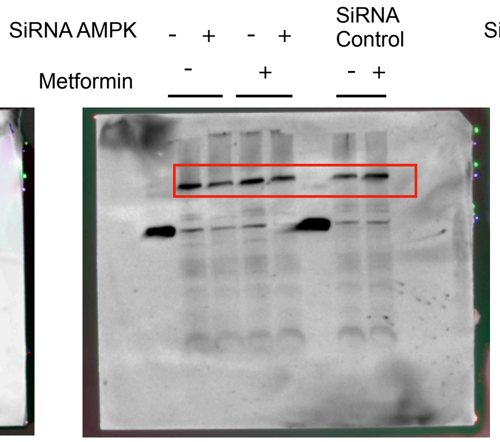

Western Blot : Sortilin SW-620 Low Glucose *in vitro*
Figure 4(e)

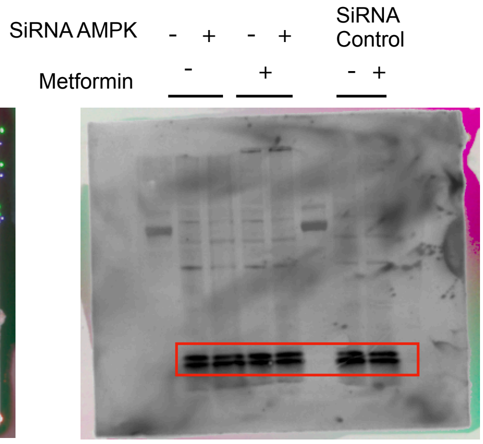

Western Blot : LC3-B SW-620 Low Glucose *in vitro*
Figure 4(e)

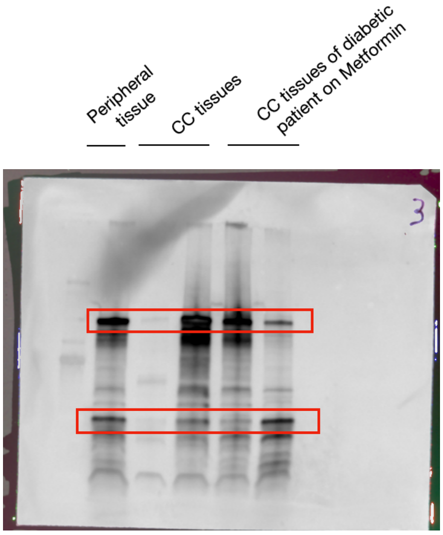

Western Blot : E-cadherin *ex vivo*, **stade I patients'** *(Figure 8(a))*

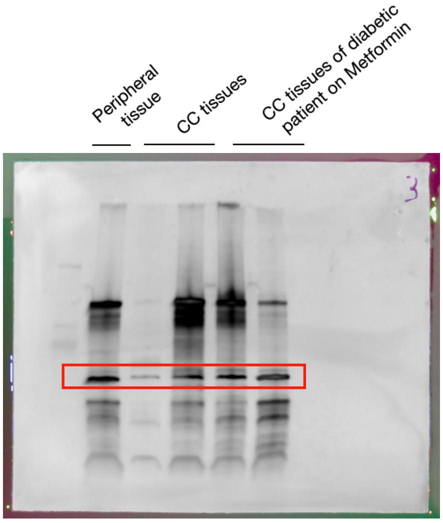

Western Blot : Actin of E-cadherin Western Blot *ex vivo*, **stade I patients'** *(Figure 8(a))*

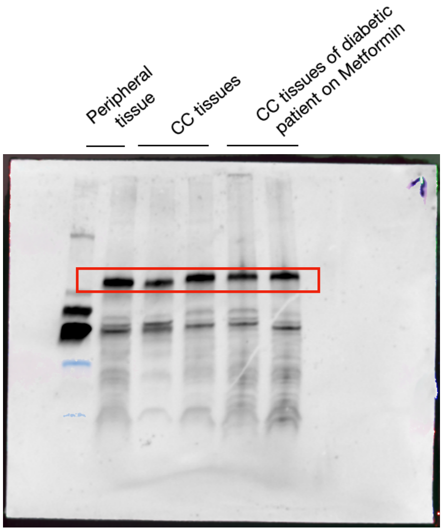

Western Blot : Sortilin *ex vivo*, **stade I patients' (***Figure 8(a))*

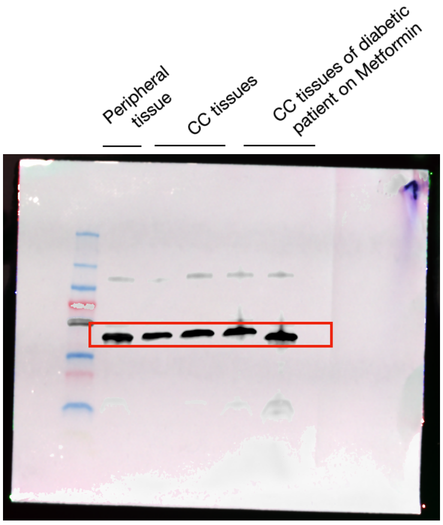

Western Blot : Actin of Sortilin Western Blot *ex vivo*, **stade I patients'** *(Figure 8(a))*

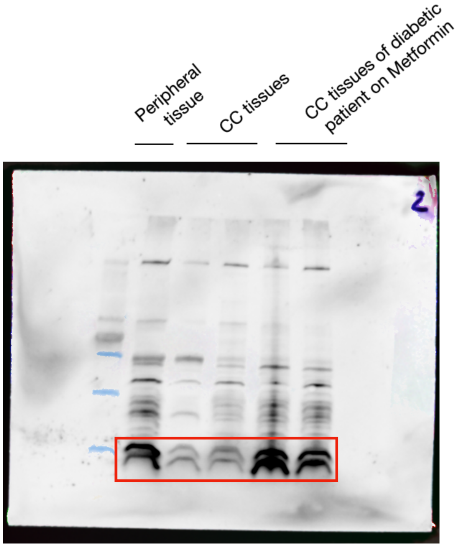

Western Blot : LC3-B *ex vivo*, **stade I patients'** *(Figure 8(a))*

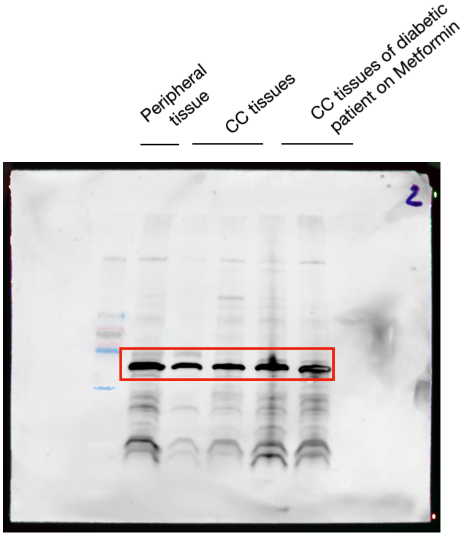

Western Blot : Actin of LC3-B Western Blot *ex vivo*, **stade I patients'** *(Figure 8(a))*

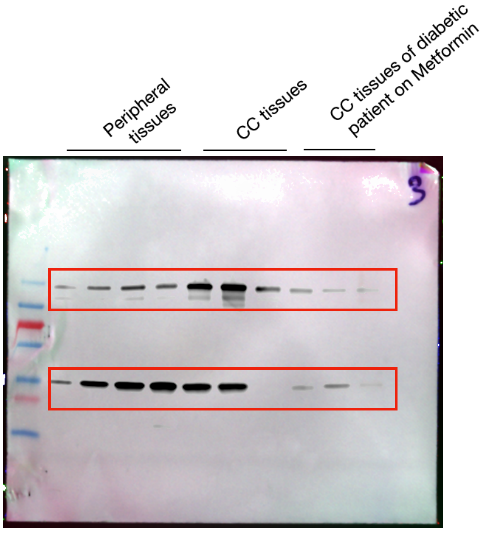

Western Blot : E-cadherin *ex vivo*, **stade II patients'** *(Figure 8(b))*

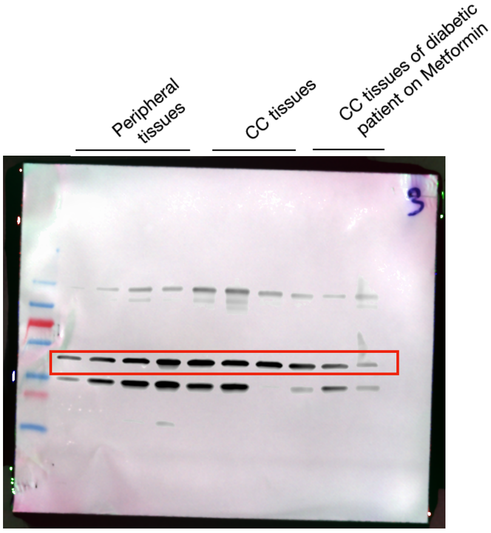

Western Blot : Actin of E-cadherin Western Blot *ex vivo*, **stade II patients'** *(Figure 8(b))*

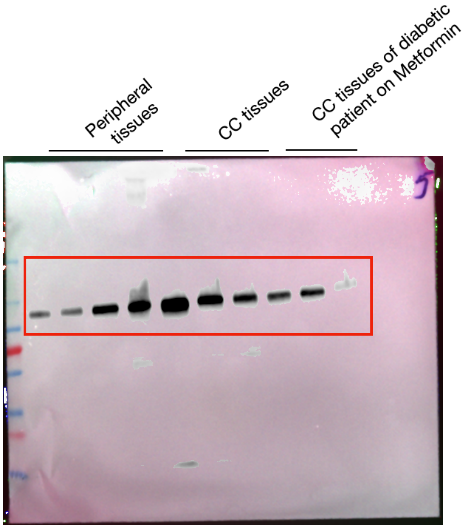

Western Blot : Sortilin *ex vivo*, **stade II patients'** *(Figure 8(b))*

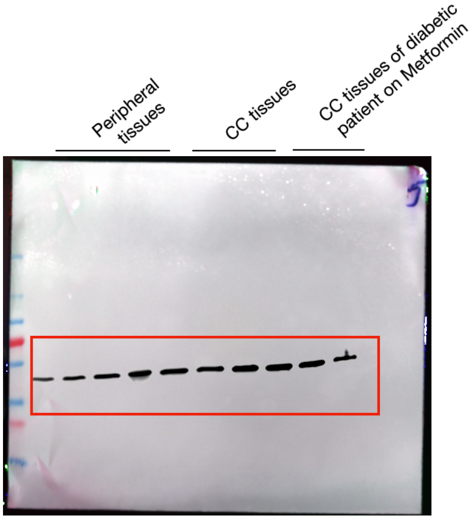

Western Blot : Actin of Sortilin Western Blot *ex vivo*, **stade II patients'** *(Figure 8(b))*

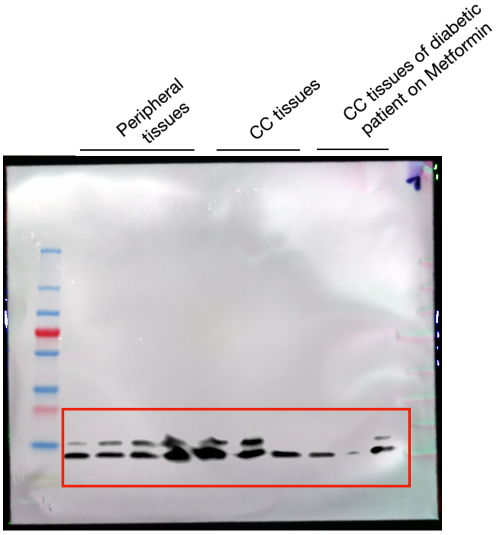

Western Blot : LC3-B *ex vivo*, **stade II patients'** *(Figure 8(b))*

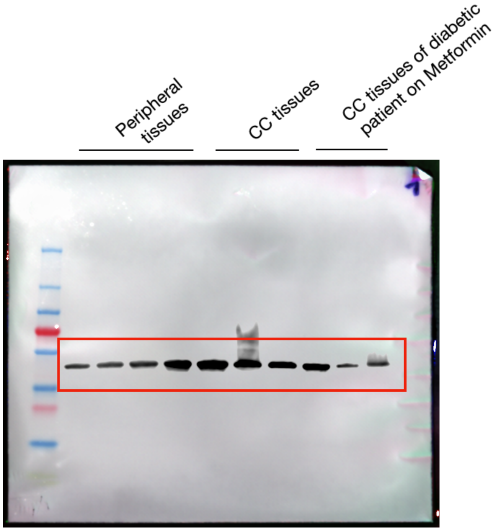

Western Blot : Actin of LC3-B Western Blot *ex vivo*, **stade II patients'** *(Figure 8(b))*

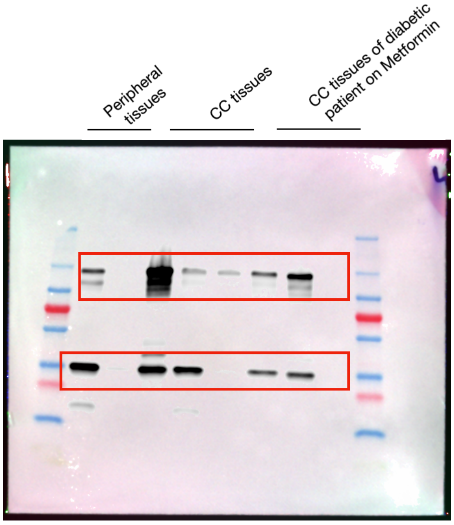

Western Blot : E-cadherin *ex vivo*, **stade III patients'** *(Figure 8(c))*

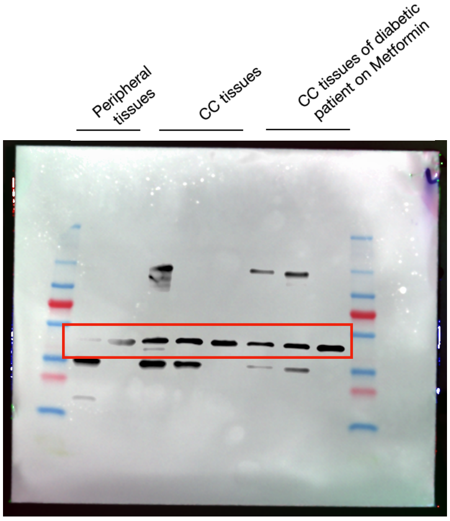

Western Blot : Actin of E-cadherin Western Blot *ex vivo*, **stade III patients'** *(Figure 8(c))*

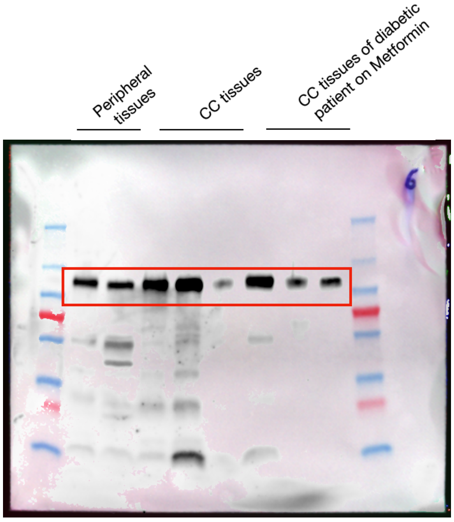

Western Blot : Sortilin *ex vivo*, **stade III patients'** *(Figure 8(c))*

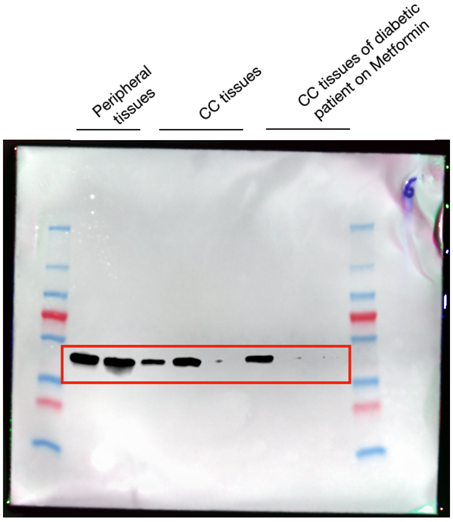

Western Blot : Actin of Sortilin Western Blot *ex vivo*, **stade III patients'** *(Figure 8(c))*

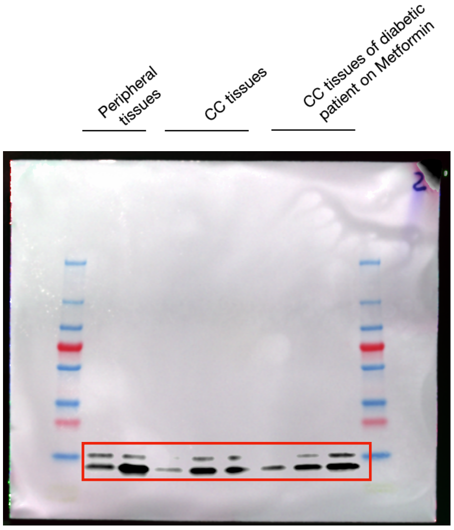

Western Blot : LC3-B *ex vivo*, **stade III patients'** *(Figure 8(c))*

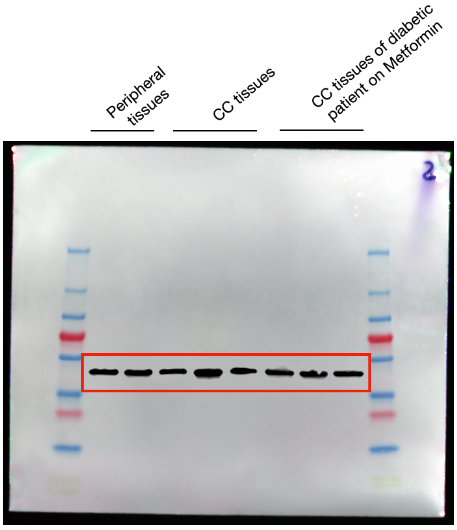

Western Blot : Actin of LC3-B Western Blot *ex vivo*, **stade III patients'** *(Figure 8(c))*
